# Do Pet Cats Deserve the Disproportionate Blame for Wildlife Predation Compared to Pet Dogs?

**DOI:** 10.3389/fvets.2021.731689

**Published:** 2021-10-25

**Authors:** Michael Franklin, Jacquie Rand, Linda Marston, John Morton

**Affiliations:** ^1^School of Veterinary Science, The University of Queensland, Gatton, QLD, Australia; ^2^Australian Pet Welfare Foundation, Kenmore, QLD, Australia; ^3^Jemora Pty Ltd., Geelong, VIC, Australia

**Keywords:** pet cat, pet dog, predation, introduced, native, wildlife, pet-related legislation

## Abstract

Concerns about the impact of pet dogs and cats on native wildlife populations have shaped pet control legislation, despite there being scant research of their impact in urban areas. Using an online questionnaire, we obtained data from 662 Australian dog and cat owners who had observed their pets capture prey in the previous 6 months. Of the pets observed to catch prey, dogs caught a median of 2 mammals, 2 birds, 2 reptiles, and 3 amphibians, whereas cats caught a median of 3 mammals, 2 birds, 4 reptiles, and 2 amphibians. Of mammals caught by dogs and cats, 88 and 93%, respectively, were identifiable as introduced mice, rats, and rabbits. Of pets that caught prey, a substantial proportion caught native animals (62% of dogs and 47% of cats). However, median numbers of native animals caught per dog (2) or cat (3) over 6 months were low. Small skinks and lizards comprised the greatest proportion for dogs and cats, but dogs also caught larger native prey (e.g., possums, kangaroos, and wallabies). Most birds caught by dogs and cats were common or introduced (dogs: crested pigeons and lorikeets; cats: noisy miners and rosellas). To design measures that will effectively protect Australia's native wildlife, thorough understanding of the role dogs and cats play in Australian urban ecosystems is required. These findings can inform that understanding, and assist with development of management strategies for urban dogs and cats, and as well as directing resources to efforts that will most protect urban wildlife.

## Introduction

The introduction of non-native species to a new region can have significant impacts on local ecosystems. Introduced species can alter ecosystem dynamics in a number of ways, such as through increased competition for resources and predation of native species (1, 2). Domestic dogs (*Canis familiaris*) and domestic cats (*Felis catus*) have been introduced to a variety of ecosystems globally and hunt successfully in many of them (3–5). The knowledge of what pet dogs and cats are hunting and the numbers and proportion of their prey that are native species is a guide to their impact on wildlife populations in urban areas. This information is useful for developing effective management strategies and directing resources in urban areas to protect native species, particularly given that the approach may differ between disturbed urban and undisturbed natural environments.

Feral cats have played a role in the extinction of native species in mainland Australia and on Australian islands (6, 7). However, while feral cats usually live in fairly remote, undisturbed habitats, and must hunt for all their food, cats in urban and peri-urban areas live in locations where native habitats are highly disturbed by humans, and they generally rely on humans intentionally or unintentionally to provide most or all of their food (8, 9). A recent Australian study reported that predation rates of individual pet cats are about 25% that of feral cats, but that densities of pet cats are much greater than feral cats (10). For cats in particular, a lack of understanding surrounding their impact on native wildlife in urban areas has hindered the development of more effective management strategies. The strategies available to manage owned, semi-owned, and unowned cats in urban areas, where cats typically have a direct or indirect association with humans, differ greatly from those that can be used to control feral cats in undisturbed habitats where the cats do not have a relationship with humans, and rely solely on prey for survival.

In urban areas, the regulations for dogs typically involve requiring them to stay confined to the owner's yard and walking them on a leash in certain areas. In the case of cats, the precautionary approach is often employed, with the aim of preventing pet cats from hunting (11). This has led to various local municipal councils introducing regulations such as designated cat-free zones, requirements for cats to wear bells, dusk to dawn curfews, and requirements to keep cats inside at all times (11, 12). Although cat owners are subject to legislation regarding containment, in urban and peri-urban areas, between 0.7 and 1.5 million stray cats are fed by people who do not perceive they own them (13, 14). In many countries other than Australia, trap–neuter–return is used as a method to control urban stray cats and involves desexing them and returning them to the location where they were caught, preferably with an identified carer (15–19). However, this strategy is illegal in Australia, and public concerns regarding effects on native wildlife have hindered trials of this management approach (20), despite evidence they can decrease cat numbers under certain circumstances, and when performed with sufficient intensity (14, 21, 22). Hence, it can be seen that the uncertainty surrounding the impacts of cats on native wildlife are currently impacting upon the design and implementation of methods to control cats in urban areas (5, 23, 24). While a few studies have been undertaken to examine the impact of predation by owned cats (25–29) and owned dogs (28, 30–34), they have typically examined the impact of predation by these species separately, so the results are not directly comparable between dogs and cats.

The primary aims of our study were to describe animal prey that owners observed to be caught by their pet dogs and cats in Australia, to compare these between dogs and cats, and to determine what proportion of these prey are native animals. This information gathered in the survey could be utilized to direct and develop more effective management strategies for dogs and cats in urban areas to better protect Australian native wildlife.

## Materials and Methods

### Study Overview

**Figure 1:**
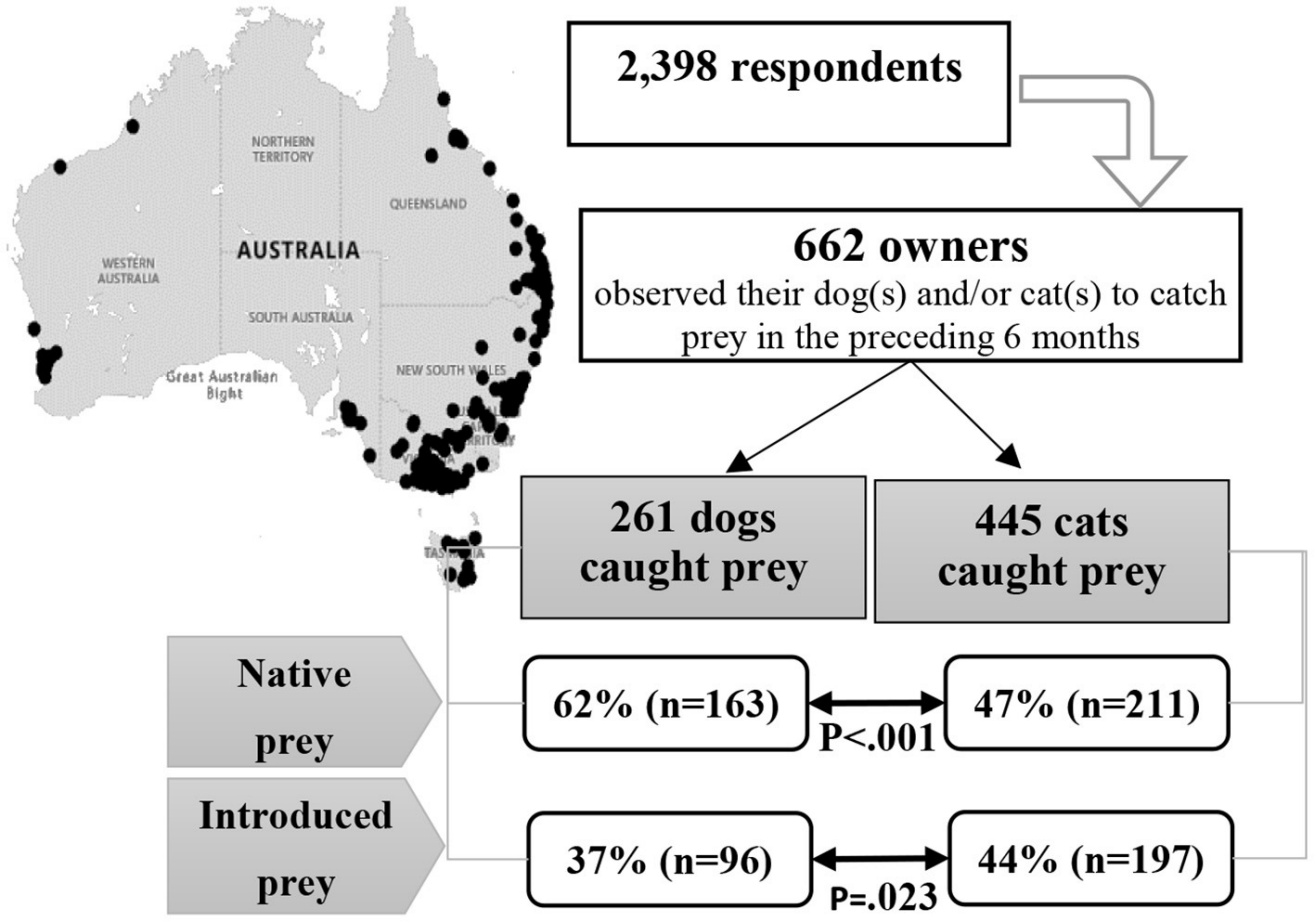
Flowchart showing total number of respondents, number of owners that observed their dog(s) and/or cat(s) to catch prey in the preceding 6 months, numbers of dogs and cats observed to catch prey, and the percentages of preying dogs and cats that caught native and introduced prey. *P* values are for comparison between dogs and cats for the given prey type; in addition, for 40% of these 445 cats and 25% of these 261 dogs, the description of some or all of the species they caught was insufficient to allow classification as native or introduced species. For some cats and dogs, none of the species they caught could be classified as native or introduced species.

We conducted a survey with self-selected dog and cat owners providing data through a questionnaire administered using an online survey software package. We asked dog and cat owners to participate in the study and informed them that by completing the questionnaire, they would be contributing to knowledge about the hunting behavior of domestic dogs and cats. They were also informed that this information would assist in making recommendations as to what sort of action will effectively help to protect native wildlife. Respondents were also asked about pet containment and attitudes to predation (not reported here). Respondents were not required to own a pet that caught prey, or a pet that had the opportunity to hunt. For the study reported here, only respondents whose dogs and/or cats had caught animal prey were selected for analyses. An overview of the study is presented in Figure 1. The study was approved by The University of Queensland Human Ethics Committee (approval number 2014000597).

### Questionnaire Design

A questionnaire (Supplementary Table 1) was created using online survey software developed by Qualtrics (Qualtrics, Provo, UT, USA). Responses (*n* = 299) to a previous online pilot questionnaire (conducted in 2014) were used to refine the design of this questionnaire. The questionnaire collected information including basic demographic data about the respondent, details of their pet's age, sex, and breed, information regarding each pet's outdoor environment, how often the respondent had observed each of their pets to hunt over the previous 6-month period (from memory), and details of what their pet had hunted over this time.

### Enrollment of Dog and Cat Owners

Australian pet-related organizations were asked to assist with distributing the questionnaire to the public by publishing a link to the questionnaire through their webpages, newsletters, and/or Facebook pages. These organizations included the RSPCA, Dogs Queensland, Getting to Zero, Cats of Australia, and many others. Members of the public could then voluntarily elect to complete the questionnaire. Respondents were eligible only if they were aged 18 years or more, were Australian residents, and they currently owned at least one dog or cat. Responses were received from July to November 2015.

### Classification of Prey Animals

Each reported prey animal was classified into one of the following three groups: (1) native, (2) introduced or domestic, or (3) unable to be classified. Several rules were created to further classify prey animals (Table 1). Postcode, property type, the respondent's description of the prey, and advice from experts from the Australian Museum (personal communication H. Cogger) and The University of Queensland (personal communication P. Murray) were used to assist with the classification of prey animals. One cat was reported to have caught goldfish, but this prey type was not included in data analyses. Insect prey was also disregarded. In the text, the term “unidentifiable” is used where it was known, for example, that it was a rodent, but it could not be determined from the description if it was a mouse, a rat, or another type of rodent. Similarly, “unclassifiable” was used when it was unknown if it was introduced or native, for example, a rodent might have been identifiable as a rat, but it could not be classified as introduced or native. The CSIRO List of Australian Vertebrates was used as a point of reference for the genus and species names presented in the Tables A1–A4 (35).

**Table 1:** Rules applied for classifying prey as native, introduced, or unable to classify.

**Category**	**Description**
Mammals	Based on personal communication with Peter Murray, The University of Queensland, the following rules were applied:
	1. Rats and mice were classified as introduced except in rural areas or areas near environment considered relatively undisturbed by humans, where they were classified as unknown unless the respondent's description allowed identification.
	2. Other mammals were easily classified based on the respondent's description.
Birds	The Reader's Digest Complete Book of Australian Birds was used as the final point of reference. The following rules were applied:
	1. If the respondent specified a species, its geographical range was checked against the respondent's postcode to confirm it was a plausible prey animal in that area. If so, the respondent was assumed to have identified the animal correctly.
	2. When the respondent provided only a description of the bird, all possible species consistent with this description found near the respondent's postcode were determined. The prey animal was then classified as follows:
	a. Native if:
	i. One or more of these possible species is native and common in the area, AND
	ii. One or fewer of these possible species is not native and uncommon to rare in the area.
	b. Introduced if:
	i. One or more of these possible species is not native and common in the area, AND
	ii. One or fewer of these possible species is native and uncommon to rare in the area.
	c. Unable to classify if the bird did not fit either of these rules.
Reptiles and amphibians	The following information was obtained from the Australian Museum (personal communication Hal Cogger) and The University of Queensland (personal communication Peter Murray): *In most parts of Australia, any small lizard is likely to be native, except in the tropics/subtropics where two introduced gecko species are common*. Hence, the following rules were applied:
	1. Any small lizard not caught within the range of these two gecko species was classified as native.
	2. Any gecko caught within the range of these two geckos was considered not native unless the respondent's description suggested otherwise.
	3. For any prey described as a “small lizard” or “skink” within the range of these geckos, it was classified as native if an accompanying description was indicative of a lizard other than a gecko, or if the respondent had separately reported capture of geckos. Otherwise, it was classified as unknown.
	4. Other reptiles and amphibians were easily classified based on the respondent's description and postcode.

### Statistical Analyses

The individual pet (dog or cat) was the unit of analysis. Among the animals that had caught prey, proportions that had caught each type of prey were compared between dogs and cats. For each type of prey, each dog and cat were classified as either having caught or not having caught that prey. Distributions of these binary variables were compared between dogs and cats using likelihood ratio test *p*-values from logistic regression models with respondent fitted as a random effect to account for clustering of pets within respondents. Where five or fewer dogs and/or cats caught the prey type, Fisher's exact tests were used instead of logistic regression models. These were calculated using, respectively, the -xtlogit- and -tabi- commands in Stata (version 15, StataCorp, College Station, Texas, USA). Interactions between pet species (dog or cat) and each of state and property type in their effects on whether preying dogs and cats caught native prey were also assessed using random effects logistic regression. For interactions with state, only dogs and cats from New South Wales, Queensland, and Victoria were included as other states and both territories had few preying dogs and cats in the study. For the same reason, for property type, only dogs and cats from residences with garden and acreage/semi-rural/farm were included. *p*-values for all interaction terms jointly were assessed using likelihood ratio test *p*-values. The same interactions were also assessed in their effects on whether preying dogs and cats caught introduced prey using the same methods.

For each type of prey, the reported numbers observed to be caught by each dog and cat in the previous 6 months were also analyzed. Distributions of numbers of that prey type caught in the previous 6 months per pet were compared between dogs and cats that had caught that prey type using Mann–Whitney (Wilcoxon) rank-sum tests calculated with Stata's—ranksum—command. The Fisher's exact tests and Mann–Whitney (Wilcoxon) rank-sum tests did not account for clustering of pets within respondent, but more complex statistical methods were considered inappropriate given the limited numbers of pets for many of these comparisons and the low mean number of pets per respondent (706/552 or 1.3). Two-tailed *p*-values were used. Data for unclassified prey (i.e., those where it was not known whether they were native or introduced) were not compared statistically between dogs and cats. Each of these would have been either native or introduced. As such, there were no generalizable hypotheses to test statistically for these prey.

For all reported data, prey reported to be caught refers to prey observed by owners to be caught by their pets in the previous 6 months. We have compared proportions of dogs and cats that caught each type of prey among dogs and cats that had caught prey. We did not study which of all dogs and all cats (i.e., preying and non-preying combined) are more likely to catch each prey type (see Discussion on limitations).

**Figure A1:**
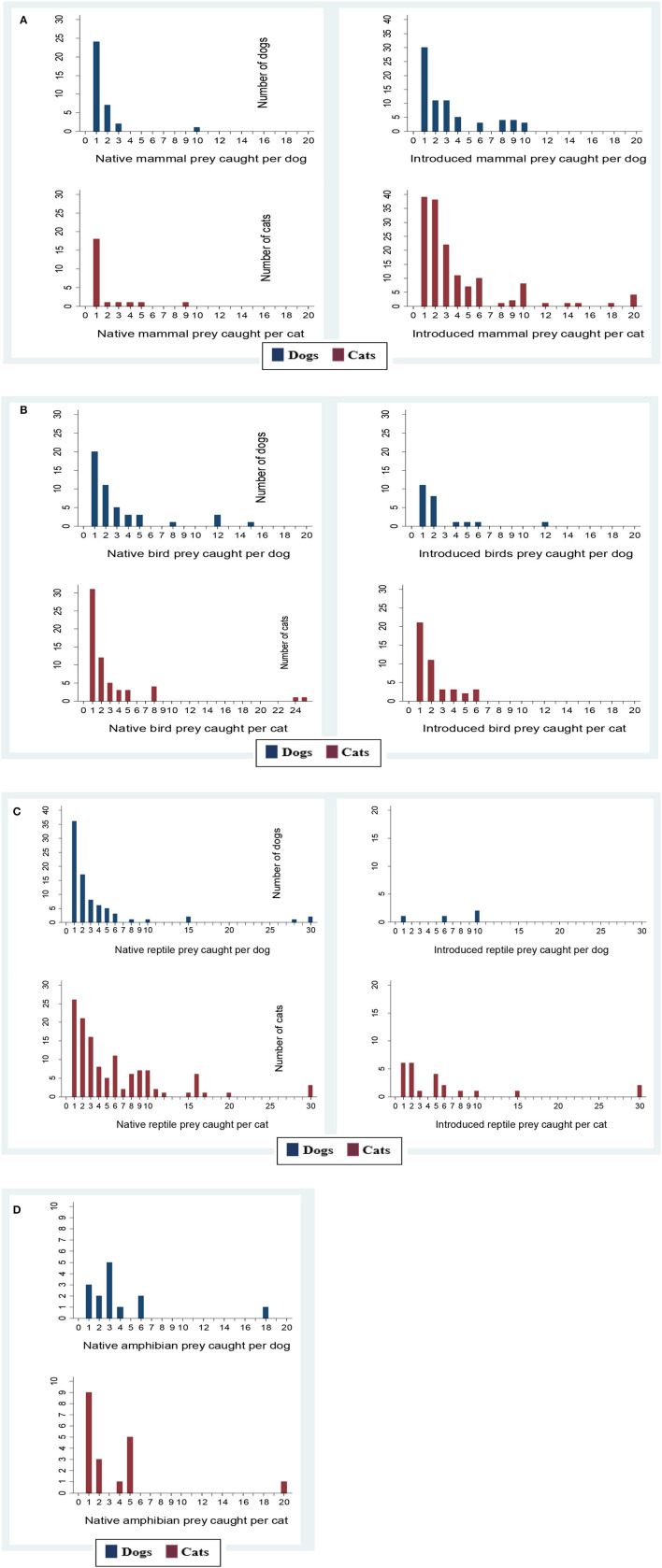
**(A–D)** Distribution of numbers of native and introduced prey caught per dog or cat for the 1,375 and 3,874 prey animals reported to have been caught by 261 dogs and 445 cats, respectively, in the 6 months immediately preceding when their owners completed the study questionnaire. **(A)** Distribution of mammal numbers caught per dog or cat. **(B)** Distribution of bird numbers caught per dog or cat. **(C)** Distribution of Reptile numbers caught per dog or cat. **(D)** Distribution of Amphibian numbers caught per dog or cat.

In the Results section, the median numbers of prey caught were used in favor of means as the measure of central tendency, as numbers caught were highly right-skewed and means would be markedly influenced by the high reported values, many of which appear to be only approximate estimates given the frequency of rounding to the nearest 10 for many high values. In addition, as these numbers are highly right-skewed (Figure A1), medians provide a better estimate of the typical numbers observed to be caught by any particular individual prey-catching dog or cat. Ranges and distributions are provided as an indicator of the maximum number and distribution of the numbers of particular prey caught per dog or cat. Median number, range, and distribution of prey caught are shown only for dogs or cats that caught that particular prey type.

The results must be interpreted considering the proportion of prey that could not be classified. Where there are a relatively high proportion of “unknowns” [e.g., for mice (*Mus musculus*) and rats (*Rattus*), and to a lesser extent for birds], it renders the relative proportions of the remaining “knowns” less definitive. For instance, at least in theory, the higher proportion of dogs (than cats) capturing known native species could be reduced if for some reason most of the unknown mammals preyed on by cats were native.

## Results

### Respondents

Of the 2,398 responses received, ineligible respondents were sequentially excluded because they did not record their age or recorded an implausible value (*n* = 176), were aged <18 years (*n* = 8), were not an Australian resident (*n* = 28), had entered an invalid Australian postcode (*n* = 3), and did not nominate that they currently owned a dog or cat (*n* = 68). Of the remaining 2,115 respondents, 847 (40%) nominated that they had seen evidence that one or more of their currently owned dog(s) and/or cat(s) had caught prey animals in the 6 months immediately preceding when they completed the questionnaire (24% or 364 of 1,529 dog owners and 41% or 550 of 1,346 cat owners). Of these 847 respondents, 662 provided details of the number and type of prey caught for specific dogs or cats that had caught at least one prey in the past 6 months, so were eligible and were enrolled in the study.

Most respondents were female (90%), the majority lived in either Queensland, New South Wales, or Victoria (78%), and the majority lived in a residence with a garden (71%; Table 2, Figure 1). The ≥56 years age group (17%) was under-represented compared with the wider Australian population, considering that the 2016 census conducted by the Australian Bureau of Statistics shows that ~35% of Australian adults were over 55 years of age. Each of the other age groups was slightly over-represented.

**Table 2:** Demographic details of 662 respondents who owned dogs or cats reported having caught prey in the 6-month period immediately preceding when they completed the study questionnaire.

**Category**	**Response**	**Respondents**	
		**%**	**(*n*)**
Sex	Male	10	(65)
	Female	90	(597)
Age	18–25	16	(107)
	26–35	23	(154)
	36–45	23	(156)
	46–55	20	(133)
	≥56	17	(112)
State/territory	New South Wales	27	(177)
	Australian Capital Territory	9	(60)
	Northern Territory	1	(5)
	Queensland	23	(153)
	South Australia	3	(22)
	Tasmania	4	(29)
	Victoria	28	(187)
	Western Australia	4	(29)
Property type	Residence without garden	3	(17)
	Residence with garden	71	(468)
	Farm, acreage or semi-rural	25	(165)
	Other	2	(12)
Respondents who owned only dog(s) reported to catch prey		35	(235)
Respondents who owned only cat(s) reported to catch prey		57	(377)
Respondents who owned both dog(s) and cat(s) reported to catch prey		8	(50)

### Details of Prey Caught

Of the 662 respondents, 285 provided details (including details of the number of prey caught) for specific dogs that had caught at least one prey (excluding insects) in the preceding 6 months, and 427 provided details for specific cats that had caught at least one prey (excluding insects and fish) in the preceding 6 months, including 50 respondents who provided data for at least one dog and one cat. In total, 388 dogs and 555 cats were reported to have caught at least one prey animal in the past 6 months.

Respondents provided sufficient detail about specific prey types to allow classification (and included estimated numbers caught) for 1,757 prey observed to be caught by dogs in the past 6 months, including 1,375 where the capture was attributed to a specified dog, and for 4,367 prey observed to be caught by cats, including 3,874 where the capture was attributed to a specified cat. These 1,375 prey were caught by 261 dogs owned by 222 respondents (mean of 5.3 prey per dog that caught prey) and these 3,874 prey were caught by 445 cats owned by 368 respondents (mean of 8.7 prey per cat that caught prey) (Figure 1).

#### Mammals

Mammals were the most common category of prey, and of pets that had caught prey in the preceding 6 months, 57% of cats and 47% of dogs had caught mammals (Table 3). For pets that caught mammals, the median number of mammals caught per dog in the past 6 months was 2 (range 1–81) and per cat was 3 (1–58; Table 3; Figure A1).

**Table 3:** The percentage and (number) of pets reported to catch each specified prey type for the 261 dogs and 445 cats observed to catch prey in the preceding 6 months, and median number and range caught per dog or cat in that period.

		**Percentage and (number) of pets reported to catch specified prey^a^**		**Dogs–cats comparison (*p*-value)^b^**	**Prey caught per pet: median (range)^c^**		**Dogs–cats comparison (*p*-value)^d^**
		**Dogs (*n* = 261)**	**Cats (*n* = 445)**		**By dogs**	**By cats**	
**(A) Mammals**							
Possums	Native	**10 (27)**	**3 (13)**	**0.004**	1 (1–10)	1 (1–3)	0.566
Rabbits	Introduced	**13 (33)**	**8 (34)**	**0.021**	3 (1–10)	2.5 (1–30)	0.721
Rodents	Native	0 (0)	0 (0)				
	Introduced	**12 (32)**	**26 (117)**	**<0.001**	2 (1–80)	2 (1–40)	0.184
	Unclassified	12 (31)	25 (110)		2 (1–30)	4 (1–52)	
Bats	Native	0.4 (1)	0.7 (3)	1.000	1 (1–1)	1 (1–2)	0.564
Domestic		**3 (8)**	**0 (0)**	**<0.001**	1 (1–4)		
Other	Native	2(6)	2 (9)	0.806	1.5 (1–3)	1 (1–8)	0.789
	Introduced	**2 (5)**	**0 (0)**	**0.007**	1 (1–2)		
Pooled	Native	**13 (34)**	**5 (23)**	**0.002**	1 (1–10)	1 (1–9)	0.728
	Introduced^e^	28 (73)	33 (149)	0.124	2 (1–80)	2 (1–40)	0.15
	Unclassified	12 (31)	25 (110)		2 (1–30)	4 (1–52)	
All types		**47 (123)**	**57(253)**	**0.032**	**2 (1–81)**	**3 (1–58)**	**<0.001**
**(B) Birds**							
Columbidae	Native	3(8)	1 (5)	0.092	2(1–15)	3 (1–24)	0.652
	Introduced	2 (5)	2 (7)	0.734	2 (1–6)	1 (1–5)	0.662
	Unclassified	4 (11)	4 (18)		2 (1–3)	2 (1–18)	
Passerines	Native	8(20)	10 (46)	0.173	1.5 (1–6)	1 (1–25)	0.915
	Introduced	**2(6)**	**8 (35)**	**0.011**	3 (1–12)	2 (1–6)	0.240
	Unclassified	4(10)	5 (24)		2 (1–6)	2(1–10)	
Psittacines	Native	7 (18)	4 (18)	0.469	2.5 (1–12)	2 (1–40)	0.456
	Unclassified	1 (2)	1 (3)		1 (1–1)	2 (2–4)	
Wild fowl	Native	2 (5)	0 (0)	**0.007**	2 (1–3)		
	Introduced	0.4 (1)	0 (0)	0.370	1 (1–1)		
	Unclassified	1 (2)	0.4 (2)		1 (1–1)	16 (2–30)	
Domestic		**4 (11)**	**0.3 (1)**	**<0.001**	1 (1–2)	2 (2–2)	0.317
Other	Native	**1 (3)**	**0 (0)**	**0.050**	1 (1–1)		
	Unclassified	4 (11)	6 (28)		1 (1–10)	2 (1–10)	
Pooled	Native	18 (47)	14 (62)	0.360	2 (1–15)	1.5 (1–40)	0.597
	Introduced^e^	9 (23)	10 (43)	0.688	2 (1–12)	2 (1–6)	0.919
	Unclassified	13 (35)	16 (72)		2 (1–10)	2 (1–30)	
All types		36(94)	35 (156)	0.848	2 (1–18)	2 (1–40)	0.512
**(C) Reptiles**							
Geckos	Native	0 (0)	0.4 (2)	0.533	–	1.5 (1–2)	–
	Introduced	**2 (4)**	**5 (24)**	**0.01**	8 (1–10)	2.5 (1–30)	0.351
	Unclassified	0.4 (1)	3 (12)	**–**	1 (1–1)	2.5 (1–6)	–
Large lizards	Native	**18 (46)**	**4(16)**	**<0.001**	1 (1–10)	1 (1–30)	0.768
Lizard (size unknown)	Native	2 (4)	1 (5)	0.732	2 (1–30)	3 (1–5)	0.800
Small lizards/skinks	Native	10 (27)	25 (109)	**0.001**	4 (1–40)	5 (1–150)	0.104
	Unclassified	0.4 (1)	0 (0)		5 (5–5)	–	–
Snakes	Native	3 (7)	3 (14)	0.747	1 (1–5)	1 (1–4)	0.925
Other	Native	1 (2)	0 (0)	0.136	1.5 (1–2)	–	–
Pooled	Native	32 (83)	30 (134)	0.16	**2 (1–40)**	**4 (1–150)**	**<0.001**
	Introduced	**2 (4)**	**5 (24)**	**0.01**	8 (1–10)	2.5 (1–30)	0.351
	Unclassified	1 (2)	3 (12)	–	3 (1–5)	2.5 (1–6)	–
All types		34 (88)	36 (162)	0.759	**2 (1–40)**	**4 (1–150)**	**<0.001**
**(D) Amphibians**							
Frogs	Native	5 (14)	4 (19)	0.465	3 (1–30)	2 (1–20)	0.189
Toads	Introduced	0.4 (1)	0 (0)	0.370	12 (12–12)		
	Unclassified	0 (0)	0.2 (1)			2 (2–2)	
Pooled^b^	Native	5 (14)	4 (19)	0.465	3 (1–30)	2 (1–20)	0.189
	Introduced	0.4 (1)	0 (0)	0.37	12 (12–12)		
	Unclassified	0 (0)	0.2 (1)			2 (2–2)	
All types		5 (14)	4 (20)	0.586	3 (1–30)	2 (1–20)	0.114

*Only dogs and cats where the respondent attributed the capture of at least one identifiable prey in the preceding 6 months to that dog or cat were included. Percentages are rounded to the nearest number. Bold indicates values are significantly different (p < 0.05) between dogs and cats*.

a *For dogs and cats that had caught that type of prey*.

b *p-value for whether pet caught this prey type for dogs vs. cats*.

c *Individual dogs and cats could have caught prey from more than one category. Median number and range of prey caught are shown only for dogs or cats that caught that particular prey type*.

d *p-value obtained from the Mann–Whitney test comparing distribution of this prey type caught in the previous 6 months per pet between dogs and cats that had caught that prey type*.

e *ncludes domestic*.

Of cats observed to have caught prey, 51% caught rodents, most of which were likely introduced given their urban location (Table 1). No cats were observed to catch native rodents (Table 3A). Fewer caught other mammal types such as rabbits (*Oryctolagus cuniculus*; 8%). In contrast, of all dogs that caught prey, 24% caught rodents, followed by rabbits (13%) and possums (*Phalangeriformes*; 10%). Among dogs and cats that caught prey, dogs (10%) were more likely than cats (3%) to catch possums (*p* = 0.004), and more dogs (13%) than cats (5%) caught native mammals (*p* = 0.002; Table 3A).

**Figure 2:**
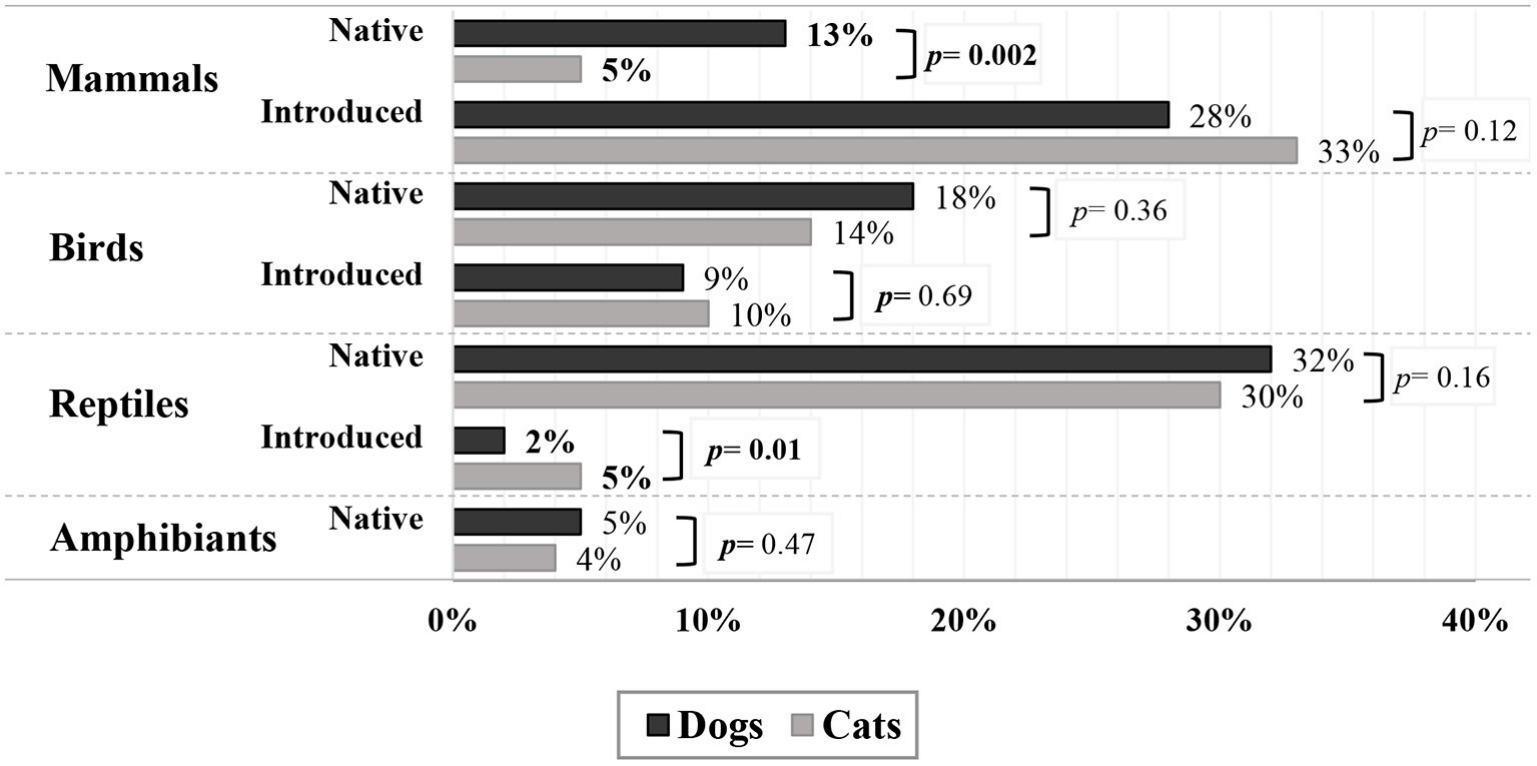
Of the 261 dogs and 445 cats that caught prey in the preceding 6 months, the percentages show the specified prey type caught; *p* values from random effects logistic regression (or where five or fewer dogs and/or cats caught the prey type, Fisher's exact tests) for whether pet caught this prey type for dogs vs. cats (of dogs and cats that caught prey in the preceding 6 months); bold indicates values are significantly different (*p* < 0.05) between dogs and cats.

Cats caught 1,523 mammals in the past 6 months, of which 53% were mice, 28% were rats, 1% were either mice or rats (it was not possible to identify which), and 12% were rabbits, accounting for 93% of mammals caught by cats (with a further 4% unidentifiable; Table A1). Native animals comprised only a small proportion of mammals caught [including possums 1%, sugar gliders (*Petaurus breviceps*) 0.6%, and bandicoots (*Peramelemorphia*) 0.3%; Table A1 and Figure 2]. Dogs caught 626 mammals, of which 44% were rats, 23% mice, 1% were either mice or rats, and 20% were rabbits accounting for 88% of mammals caught (1 mammal was unidentifiable). Only a small proportion was native species (including possums 7%, kangaroos (*Macropodidae*) or wallabies (*Notamacropus*) 1%, bandicoots 0.3%; Table A1).

#### Birds

Of all pets observed to have caught prey in the last 6 months, 36% of dogs and 35% of cats caught birds (Table 3). For pets that caught birds, the median number of birds caught in the past 6 months was 2 (range 1–18) for dogs, and 2 (1–40) for cats (Table 3B; Figure A1). For cats, this median of 2 was lower compared with the corresponding medians for mammals (3) and reptiles (4). Native birds were caught by more dogs (18%) and cats (14%) than were introduced birds (9% of dogs and 10% of cats). More dogs (4%) than cats (0.3%) were reported to catch domestic birds such as chickens (*Gallus gallus domesticus*). Of pets that caught prey, native passerines were caught by only 8% of dogs and 10% of cats, and relatively few caught native psittacines (7% of dogs, 4% of cats; Table 3B).

Of classifiable birds caught by cats, the greatest proportion were passerine and were most commonly noisy miners (*Manorina melanocephala*), followed by sparrows (*Passeridae*) and equal proportions of Indian mynas (*Acridotheres tristis*), starlings (*Sturnidae*), and fairy wrens (*Malurus*; Table A2). Psittacines were less frequently caught and were most commonly rosellas (*Platycercus*) and red-winged parrots (*Aprosmictus erythropterus*). Similarly, for dogs, the greatest proportion was passerine and was most commonly starlings and magpies (*Gymnorhina tibicen*). Of birds caught by dogs and cats, more were native (Figure 2).

#### Reptiles

Of pets observed to have caught prey, 34% of dogs and 36% of cats caught reptiles (Table 3). These are similar to the percentages of dogs and cats reported to catch birds. For pets observed to catch reptiles in the previous 6 months, the median number caught per dog was 2 (range 1–40) and per cat was 4 (1–150), but the range was large, indicating that some individual dogs and cats were more prolific hunters than others (Table 3C; Figure A1). Of dogs and cats, similar proportions caught native reptiles (32% for dogs, 30% for cats). Of all cats that caught prey, 25% caught small native lizards or skinks (*Scincidae*), whereas only 10% of dogs had caught these small lizards, with a higher proportion (18%) catching larger reptiles such as blue-tongued skinks (*Tiliqua*) and/or snakes. Of reptiles caught by cats, 97% were small lizards and skinks or Asian geckos (*Hemidactylus frenatus*; Table A3). Of reptiles caught by dogs and cats, most were native (Figure 2).

#### Amphibians

Amphibians were the least common type of observed prey, caught by only 5% of dogs and 4% of cats observed to catch prey. Most amphibians caught by both dogs and cats were native frogs. Dogs that caught amphibians caught a median of 3 (range 1–30) amphibians, and cats caught 2 (1–20; Table 3D; Figure A1).

### Native vs. Introduced Prey

Among preying pets, more prey animals were reported to be caught in the past 6 months per cat than per dog with medians of 4 (range 1–155) for cats and 2 (range 1–81) for dogs (*p* < 0.001). The difference was largely due to the number of small lizards and introduced mice and rats caught by cats.

Overall, among pets observed to have caught prey, a larger proportion of dogs (62%) caught native prey than cats (47%; *p* < 0.001). When interaction terms were fitted, there was some evidence that the odds of preying dogs catching native prey relative to that for cats differed by state (*p* for interaction terms jointly 0.097), with dogs much more likely than cats to catch native prey in New South Wales and Queensland (*p* = 0.013 and 0.007, respectively), while in Victoria, the observed odds of dogs and cats catching native prey were similar (*p* = 0.847). There was no compelling evidence that the odds of preying dogs catching native prey relative to that for cats differed by property type (*p* for interaction term 0.249). Overall, a larger proportion of preying cats caught introduced prey (44%) than dogs (37%; *p* = 0.023; Table 3), but this varied with property type (*p* for interaction term 0.010). On acreage/semi-rural/farm properties, the observed odds of dogs and cats catching introduced prey were similar (*p* = 0.462) but on residences with gardens, dogs much less likely than cats to catch introduced prey (*p* = 0.001). There was no compelling evidence that the odds of preying dogs catching introduced prey relative to that for cats differed by state (*p* for interaction terms jointly 0.711).

When all native prey types were pooled, more native prey was caught per cat than per dog (median 3/cat, 2/dog; *p* < 0.001; Table 3). However, this was in large part due to the large numbers of native small lizards and skinks caught by cats. Of preying dogs and cats, 13% of dogs and 5% of cats preyed on native mammals and the median number each caught was the same (median 1/dog, 1/cat; Table 3; Figure A1). Similarly, of preying dogs and cats, 18% of dogs and 14% of cats preyed on native birds, and the median number each caught was similar (2/dog, 1.5/cat; Table 3B; Figure A1). For reptiles, 30% cats and 32% dogs preyed on native reptiles, while the median number each caught was higher for cats (4/cat, 2/dog; Table 3C; Figure A1). Of preying dogs and cats, 37% of dogs and 44% of cats preyed on introduced species, and the median number each caught was the same (median 2/dog, 2/cat; Table 3; Figure A1).

Twenty-five percent of preying dogs and 40% of preying cats caught prey that were unable to be classified as native or introduced. For dogs, 13% caught birds and 12% caught mammals, and for cats, 25% caught mammals and 16% caught birds were not able to be classified. For unclassified rodents, nearly all were able to be distinguished as a mouse or a rat and were most likely introduced, given their urban location.

## Discussion

In Australia, 40% of households own an average of 1.3 dogs (total of 5.1 million pet dogs) and 27% of households own an average of 1.4 cats (a total of 3.8 million pet cats) (36). The aims of this study were to investigate prey species that Australian pet owners observed their dogs and cats catch in the previous 6 months, including the proportion of native species, and compare them between dogs and cats. This study used owner-reported observations through a voluntary online survey advertised as an investigation of predation by pets of native wildlife, which is potentially open to participation and reporting bias. Nevertheless, the findings from this survey of 662 Australian pet owners who had observed their pets to prey in the previous 6 months (24% of study dog owners and 41% of study cat owners) provide valuable information relevant to the development of future management programs for Australian pets that aim to protect native wildlife.

### Mammals

Among dogs and cats that were observed to have caught prey, the largest proportion caught mammals (47% of dogs and 57% of cats). Of the pets that preyed on mammals, dogs caught less prey than cats (median 2/dog and 3/cat), but 97% of the identifiable mammals caught by cats were mice, rats, and rabbits, whereas this was only 88% for dogs (Table A4). While approximately half of the rodents could not be classified by owners as native or introduced (Table 1), given their location, it is likely that most of those were, in fact, introduced. In disturbed habitats, introduced rodents are much more common than native rodents (personal communication Peter Murray, The University of Queensland). Consistent with the findings of our survey, analysis of the stomach contents of trapped urban cats in the City of Brisbane (Queensland) revealed that the only prey species consumed were introduced black rats (37). Rabbits were also caught but by fewer dogs and cats in our study.

The effects of predation on wildlife populations may be negative, neutral, or positive, depending in part upon the environment and prevalence of native and introduced species in the area. For example, a study investigating the demographics of a population of eastern barred bandicoots (*Perameles gunnii*) at a rubbish tip in Hamilton, Victoria, found that cats were a substantial cause of mortality among juvenile bandicoots, although for adults, the principal cause of mortality was from motor vehicles (38). In contrast, another Victorian study of southern brown bandicoots (*Isoodon obesulus*), reported that cats did not have a negative effect. This was based on the observations that the abundance of bandicoots was highest at sites with the most urbanized surroundings where cats were most prevalent and lower in nature reserves where cats were largely absent (25). A study from Albany, New York, USA used scent stations to estimate the density of domestic cats roaming in a suburban nature reserve and compared this with the small mammal density and biodiversity within the reserve, as determined using tracking tubes. They found no link between cat density and local small animal abundance or biodiversity (39). The predation of introduced mammals may, in some situations, have beneficial effects on native wildlife populations through the suppression of some introduced species (40). For example, following eradication of cats from Little Barrier Island in New Zealand, Pacific rat (*Rattus exulans*) numbers increased, and there was a subsequent increase in predation of seabirds by rats (41). Similarly, on Australia's Macquarie Island, the eradication of cats resulted in increased rabbit, rat, and mice numbers and consequent habitat destruction (42). The effects of removing domestic dogs and cats on wildlife populations are more difficult to quantify, and the authors could find no studies where this has been undertaken in urban areas of Australia.

### Birds

Of the pets that were observed to have caught prey in the previous 6 months, birds were caught by 36% of dogs and 35% of cats. A higher proportion of dogs caught native birds (18 vs. 14% for cats, *p* > 0.05), but a similar proportion caught introduced or domestic birds (9% of dogs and 10% of cats). Passerines were the most common bird type caught by dogs and cats.

Although there are few reports from Australia comparing predation of pet dogs and cats, a study using data from a wildlife hospital in Victoria (33) found no significant difference in the number of birds injured by dogs (*n* = 33) and cats (*n* = 35). This is consistent with our finding that the median number of prey caught by dogs and cats were the same (2 birds). The low numbers of birds observed to be caught per cat over 6 months in our study is also consistent with the findings using video cameras on outdoor pet cats in New Zealand (37 cats over 3 days) and South Africa (20 cats over 5 weeks), where either no birds or only one bird was observed to be caught in the respective studies.

In our study, cats primarily caught smaller birds including Indian mynas and noisy miners. Indian mynas are introduced species common in urban areas. They are classed as a pest as they threaten native biodiversity due to their territorial behaviors and competition for nesting locations (43, 44). Although noisy miners are native, they are thriving in many urban locations and have been encroaching into the woodland habitat of some smaller native birds, which has resulted in institution of noisy miner culling programs in some locations (45, 46).

Although we did not look at the effect of predation on bird populations, owned dogs and cats may be having minimal effect on native bird populations in urban areas, given the low proportions of preying dogs and cats that caught birds and the observation that most of the native species caught are common. Domestic cats have been reported to have a negative effect on native bird populations in some areas (3, 4, 24, 47, 48). However, most studies use modeling or report hypothetical calculations based on the number of birds caught to reach this conclusion, rather than assessing actual population sizes. For example, a recent systematic review reported that domestic cats in the USA kill billions of birds and mammals annually, with most mortalities due to unowned cats (4). This estimate was reached through extrapolation of predation rates from 21 studies across the USA (12) and Europe (9), in conjunction with population estimates and other demographic data. However, the reported mortality rates were likely overestimated, particularly given that the underlying assumptions of the computer modeling were questionable, as has been discussed previously (49). For example, the authors did not take into account the relatively small amount of time that the average US pet cat spends outdoors (≤ 8 h per day) and also used very high estimates of the domestic cat population (both of owned pets and unowned strays) which were substantially larger than that provided by the AVMA (50).

Another modeling study from New Zealand collected data on predation by 144 domestic cats over a 12-month period. Using data from owner observations of prey caught by their cat, they calculated that cats have significant impacts on prey populations, particularly birds (24). However, calculations of the city-wide catch over the 12-month period for six of the bird species assessed may have been implausible as they were either close to or greater than the assumed total urban bird populations (24).

Few Australian studies have assessed the effects of domestic cats and dogs on native bird abundance and diversity, particularly for urban areas. One Australian study that did investigate this was conducted across 57 sites in metropolitan Perth. The researchers investigated factors affecting passerine bird community composition, which was the most common bird type preyed on in our study by cats. Bird data were collected at each site, and a questionnaire distributed to surrounding neighbors to determine cat and dog density. No link was found between cat or dog density and passerine bird species richness (abundance). However, a negative correlation was found between richness of bird species and both housing density and increasing distance from bushland (and decreasing size of bushland), leading the authors to suggest that habitat destruction and degradation were the critical factors rather than cats or dogs (27).

In a study involving the placement of 20 artificial birds' nests at 24 Sydney metropolitan bushland sites, it was found that the higher the cat activity (estimated by the amount of cat feces), the less nest raiding that occurred (26, 51). The authors concluded that cats reduced nest raiding by suppressing the activity of nest raiders, such as introduced black rats. In contrast, they found a negative association between density of ground-dwelling birds and cat density, but they did not investigate possible confounding by vegetation density, a highly important factor in promoting density of ground-dwelling birds (26). Given that the median home range of pet cats during the day is ~0.5 ha [75 × 75 m] (52) and bushland immediately adjacent to houses is highly disturbed (53), this association between higher cat numbers and lower ground-dwelling birds may only reflect that both cats and disturbed bushland are found adjacent to human habitation. Future studies are needed to investigate whether cats have a measurable effect on ground-dwelling bird density and diversity when the effect of density and size of native vegetation is accounted for.

Another Australian report described analyses of results from 93 studies on the frequency of occurrence of birds in cat dietary samples, combined with an estimate of the population size of feral cats and pet cats, and the authors concluded that just over 1 million birds per day are killed by cats in Australia (54). In that study, it was estimated that across Australia, 3.5% of the bird population was killed annually by cats, with the lowest numbers killed by cats in urban areas, and the highest numbers killed by feral cats in remote areas. As the authors noted, their estimate is not easily related to population viability or conservation concern for Australian birds.

The disparity between various hypothesized or estimated effects of cats on native bird and mammal populations in urban areas is, of course, both troubling and cautionary. The most plausible explanation is that the methodologies used in studies intended to generate hypotheses and estimates of cat impacts on other species are flawed. Failure to control or account for the inaccuracies that arise when the findings of research conducted in one region or environment are extrapolated to other regions or environments with significantly different attributes is probably contributing to this disparity, as is failure to account for positive impacts of cat predation on desired species by reducing the numbers of predators of the desired species, such as cat removal of rats that prey on bird nests (51).

An important further limitation of attempts to estimate the significance of predation by cats on bird populations is that they do not always properly account for the condition of the birds (e.g., whether they are sick or might otherwise not have contributed to the next breeding cycle). The average life span of banded birds of Australian species preyed on by cats is typically 2–4 years (55). This means that ~25–50% of the population of bird species susceptible to predation by cats die annually from natural causes. Studies of bird mortality from Europe found that birds killed by cats were significantly less healthy than those killed by other forms of trauma (47, 56). A study in the city of Bristol, UK, for example, found fat and pectoral muscle mass were both lower in birds killed by pet cats compared with birds killed through collisions with windows or cars (47). Another study that used dead passerine birds found by members of the public determined that spleen size was significantly smaller in birds caught by cats (*n* = 58) than those that died from collisions with windows or cars (*n* = 477) (56). The difference in spleen size was almost one-third, and thus of considerable magnitude considering that spleen size is an accepted measure of immunocompetence, and is involved in both humoral and cell-mediated responses (57). There was some evidence that younger birds were being caught by cats, but even within juveniles, spleen size was on average 48% smaller in birds caught by cats. The inference is that cat-killed birds were in significantly poorer condition than those killed following collisions, leading the authors to conclude that predation by cats represents a compensatory rather than an additive form of mortality. This would mean that predation by cats does not cause a substantial increase in the overall mortality incidence in bird populations.

The implications of these findings for public policy are clear. If predation by cats removes unhealthy individuals unlikely to breed again, and likely to die shortly from other causes anyway, the magnitude and significance of any effect urban cats have on native bird populations is at the very least unclear. Furthermore, if the large numbers of birds killed by cats that some studies report (4, 54) are misleading, they ought not to be the basis for imposing lethal control measures on urban cats unless and until more reliable measures of the impacts of cats on wildlife numbers become available. Prospective studies are urgently needed to evaluate the impact of owned dogs and cats on wildlife numbers and diversity in urban areas. These are not technically difficult to do using motion-detecting wildlife cameras, human observers, animal traps, and more recently, drones. Notably, dogs are not banned from some suburbs in Australia while cats are, although by law dogs must be confined to the owners' property. Despite this, both dogs and cats were observed to kill a median of 2 birds in 6 months, with similar proportions of their catch being native birds (50% and 40%, respectively). However, our study did not investigate what proportion of pet dogs and pet cats' prey on wildlife and that requires a different study design. Some owners whose dogs and cats are confined entirely indoors may also not have responded to this questionnaire.

### Reptiles

The impact of predation by domestic dogs and cats on native reptile numbers remains unclear, with little research performed involving domestic pets. Of the pets in this study that caught prey, approximately one-third caught reptiles, a similar proportion to those that preyed on birds. Small lizards or skinks were the most common reptilian prey of dogs and cats in our study, followed by blue-tongued skinks (considered common) for dogs and the introduced Asian house gecko for cats. A recent Australian study estimated that 53 million reptiles were killed annually by pet cats and larger numbers by feral cats in remote arid areas (58). However, they concluded that intensive studies of individual reptile species are required to contextualize the conservation consequences of such predation. This is because population size is unknown for most Australian reptile species, mortality rates due to cats will vary across reptile species, and there is likely to be marked variation among reptile species in their capability to sustain any particular predation rate. Similar investigations have not been carried out for dogs. Clearly, further studies are needed to determine the impact of domestic pets on reptile populations and determine whether these pets are a significant factor affecting the ability of reptiles to recolonise certain areas.

### Amphibians

Amphibians were the least common prey of dogs and cats, a finding consistent with other research investigating prey caught by Australian domestic cats (23). Dogs and cats most commonly caught native amphibians compared to the introduced species, with various native frogs identified as being captured.

### Native Wildlife

Our results show that of pets that caught prey, a greater proportion of dogs caught native wildlife than cats (62 vs. 47%), but the median number caught by preying cats (3) was higher than for preying dogs (2). Most of the native wildlife caught are considered common based on published descriptions of distribution and abundance of native fauna (59–62). Although greater numbers of native prey were caught per preying cat (3) than per preying dog (2), a large proportion of those caught by cats were native small lizards and skinks. Cats that caught native animals caught similar numbers of native mammals and birds as dogs (median mammals 1/cat, 1/dog; birds 1.5/cat, 2/dog).

The community perception that urban cats prey on native wildlife is correct. However, the importance of owned cats and dogs on native wildlife relative to other factors should also be considered. Our findings are consistent with a study of injured native animals brought to veterinary facilities in Tasmania and Victoria. The majority of wildlife injuries and deaths were attributed to motor vehicles (76%, 1,256), with the next highest cause of injuries and death recorded for dogs (14%, 238) followed by cats at 9% (152) (63). The NSW Wildlife Rehabilitation website (64) tracking native animals rescued shows that in 2019–2020 more native animals were injured as a result of attacks by dogs (1,522) than cats (1,199). However, collision with motor vehicles (9,610) and unsuitable environments (5,122) were more common reasons for rescue. Eastern blue-tongued skinks (354) followed by ringtail possums (121) were most often associated with dog attacks whereas ringtail possums (224) were for cat attacks. Importantly, more threatened species were rescued as a result of dog attacks (120) than cat attacks (20). For large native animals in urban areas such as koalas, dogs are a significant cause of predation, although habitat destruction and injury from cars are of greater concern (64, 65).

### Pet-Related Legislation

Various local municipal councils have introduced regulations that aim to reduce wildlife predation and problems associated with roaming cats (e.g., motor vehicle accidents and urination and defecation in yards) (12). Regulations that have been introduced include designated cat-free zones, requirements for cats to wear bells, dusk to dawn curfews, or requirements to keep cats inside at all times (11, 12). To be effective in reducing the predation of native wildlife, regulations must be appropriate for the geographical area and the species that are to be protected. In NSW, only 0.5% of injured threatened species were the result of cat attacks, and in our study, only a very small minority of cats were prolific hunters (64). Therefore, rather than attempts to confine all cats through education and legislation (and associated costs to local governments for compliance), efforts might be best directed at targeted strategies in locations where there are threatened species, such as engaging with the community to identify and assist owners with containment solutions for cats which are prolific hunters, and implementing habitat restoration, exclusion fencing, or utilizing guardian animals for more targeted protection of wildlife (66). Further research is needed to examine the impact cats have on wildlife populations in urban areas and whether larger reptiles (e.g., blue-tongued skinks) and mammals (e.g., possums) would be better protected if efforts were made to reduce predation by dogs.

It is worthwhile noting that the concern over wildlife predation has shaped cat management programs and legislation in Australia. Australian citizens have high levels of concern regarding wildlife predation by pet cats, with 95% of non-cat owners and 65% of cat owners agreeing that pet cats posed a serious threat to wildlife (67). Despite the scarcity of evidence documenting a negative impact of cats on native wildlife in urban areas of Australia, such concerns have prevented trials of non-lethal management strategies such as trap–neuter–return, as the cats are not removed from the environment and may therefore continue to prey on native wildlife. Few studies have assessed the effect of pet control regulations on wildlife in Australia. However, in a hallmark study from Perth (Western Australia), the association between density and diversity of mammals in bushland was investigated adjacent to three suburbs with differing cat management legislation (28). The sites included one where cats were banned, one where they were required to be inside overnight and wear a bell during the day, and one where there were no relevant cat regulations. Numbers of the two most abundant medium-sized mammals present, brushtail possums (*Trichosurus vulpecula*) and southern brown bandicoots (*I. obesulus*), were similar across all sites. The smaller mardo (*Antechinus flavipes*), which was regarded as highly susceptible to predation by cats, was trapped mostly at the unregulated cat site. The vegetation density was greater at this site, and the authors concluded it was vegetation density rather than cats or cat management legislation that had the greatest impact on susceptible populations. The results of this study provide evidence that determining the magnitude of effects of predation by urban domestic cats can be further complicated by contemporaneous confounding factors such as habitat loss (54, 58, 68). Further research is needed to examine the complex role pet cats now occupy in Australian urban ecosystems and their impact on populations of native wildlife and invasive species. This information is vital to ensuring that the most humane and effective methods of management are used to manage domestic pets in urban areas.

### Limitations

Voluntary online surveys are a highly cost-effective way of gathering large amounts of data but are open to selection bias. A large proportion of respondents were female, which is common in surveys where respondents are self-selected (69). We sought help distributing the survey from a wide variety of pet-related organizations, which may have introduced unknown biases into the data collection. Some groups of the pet-owning public may have been underrepresented, so our study population may not be representative of all pet owners. We specifically stated to potential respondents that we were investigating predation by pets, likely resulting in overrepresentation of pet dogs and cats that prey. However, this particular bias was not of concern as we did not aim to estimate these proportions and make no inference about those proportions. Our results also provide no indication as to which of all dogs and all cats (i.e., preying and non-preying combined) are more likely to catch each prey type.

Similar to other studies estimating the impact of cats on wildlife (24, 47), this questionnaire asked owners to describe any prey observed to be caught. Given that the predation was reported from memory, not from contemporaneous observation, this can result in the classification errors and consequent bias in results, and also could be biased toward larger prey. Accurate classification of prey as native or introduced is a further challenge, and classification of all prey was not possible. Some respondents may have been reluctant to report the capture of native or endangered species. However, as the participation in the survey was anonymous, the risk of differential underreporting for this reason was reduced. The questionnaire did not ask owners to provide an indication of the total amount of time pets that were observed, which could be problematic for estimating total numbers of prey killed, although cats may prey overnight unobserved and bring prey home. Therefore, it is not possible to know how representative our data are of all prey taken by pet dogs and cats, but it provides information about the certain types of prey that are preyed on. Notably, this same methodology has been used in other publications to support the large number of animals killed by cats (10, 24, 47).

The study has likely underestimated the number of each prey type captured, as both dogs and cats may leave prey at the site of capture or consume it (39, 70, 71). Dogs are also reported to bury prey (71). One study reported that only 23% of animals caught by cats were returned to the owner's home (70), but it is unlikely that pet owners would not see evidence of hunting if predation was frequent. For example, if the probability of an owner observing a particular caught animal was 10% and their pet caught 20 animals in the 6-month study period, there would be an 88% chance of the owner detecting at least one captured animal {calculated as 1–[(1–0.1)^∧^20]}. As such, the most reliable of our data are types of prey captured, with numbers of prey captured best used as a guide to which types of prey are captured more frequently. Additionally, the reported proportions of introduced prey (presented in Tables A1–A4) would be minimally affected by underestimation of numbers of each prey type captured if the extent of underestimation was similar for the various prey types within dogs and cats, even if owners are more likely to notice prey caught by dogs than cats (or vice versa). Other methodologies using scat or stomach content analysis have similar shortfalls as only consumed prey can be detected, resulting in bias because cats preferentially eat rodents over birds (24). The use of animal-borne video cameras does not enable such large amounts of data to be collected over such a wide geographic area as in this study. Although the information gathered in this study cannot be used to judge relative abundance or estimate genuine predation rates, it provides evidence about certain types of prey that are being caught by owned dogs and cats in Australia.

## Conclusions

This study presents relevant information about the types of prey captured by owned dogs and cats in Australia, which is an indicator of their impact on urban wildlife populations. Of predatory pets, a higher proportion of dogs were observed to prey on native wildlife than cats (62 and 47%), but the median number caught in the 6-month study period was higher for preying cats (3/cat, 2/dog) largely because of the large number of small lizards and skinks caught by cats. Of preying dogs and cats, more dogs (13%) caught native mammals than cats (5%), and the median number caught was the same (1/dogs; 1/cats). For cats, 93% of the mammals were mice, rats, and rabbits (and 4% were unidentifiable), whereas for dogs, this was 88 and 7% were possums. Similar proportions of dogs (18%) and cats (14%) preyed on native birds, with a similar number caught (2/dog, 1.5/cat). Similarly, 32% of dogs and 30% of cats preyed on native reptiles, but the median number caught was higher for preying cats (4/cat, 2/dog), most of which were small lizards and skinks.

Our results suggest that the substantial attention and blame directed at domestic cats for their hunting behavior is disproportionately large compared to that directed toward domestic dogs, given that our results show that of dogs and cats that catch prey, dogs are more likely to catch native species. Although our study was not designed to determine the proportion of pet dogs and cats that catch prey, there are 34% more pet dogs in Australia than pet cats (36, 72) and more pet cats than pet dogs are confined solely indoors.

In our study, most prey animals that could be identified were considered common or introduced. These findings raise questions about the impact that owned dogs and cats have on populations of native and introduced species in urban areas of Australia. Given the high proportion of abundant native or introduced prey caught by the cats in our study and other studies (23, 73), which in our study were predominantly small lizards/skinks, mice, and rats, cats may not be having a significant negative impact on native wildlife populations in urban areas. It has been suggested that this may be because cats exterminate susceptible species rapidly after introduction (27, 29). However, the failure of studies to find a link between high cat density and low species diversity do not support this argument (25, 27, 28, 39). Given that these studies reported that increased housing density and increased distance from bushland were associated with decreased wildlife diversity and density, efforts directed at habitat restoration and conservation of remnant bushland are likely to be the most effective strategy to protect wildlife, as opposed to pet control regulations. However, it is acknowledged that the predation of even low numbers of threatened or endangered species can have a substantial effect on their population in an urban area. Further research is required to identify specific urban locations where predation by pets could potentially impact these threatened and endangered populations.

In natural environments, native wildlife die from many causes including predation by native animals and disease. The focus should be on preserving, and where indicated, restoring populations of native wildlife. From a conservation standpoint, the critical factor is the impact owned dogs and cats have on wildlife populations as a whole, rather than how many native animals they catch. It is clear from our study that they are generally catching common species suggesting that they may not be having a significant negative effect on the overall population. As others have concluded (27, 28), hunting by domestic dogs and cats appears to be of relatively minor conservation concern compared with issues such as habitat loss and urban development. Further studies are urgently needed to better determine the factors impacting upon native wildlife abundance and diversity in specific locations. In urban areas, this will help ensure that conservation efforts and resources are directed as effectively as possible, and facilitate evidence-based pet-related legislation.

## Data Availability Statement

The raw data supporting the conclusions of this article will be made available by the authors, without undue reservation.

## Ethics Statement

The studies involving human participants were reviewed and approved by The University of Queensland Human Ethics Committee (Approval Number 2014000597). The patients/participants provided their written informed consent to participate in this study.

## Author Contributions

JR conceived the study. MF and JR developed the study design. MF, JR, and LM designed the questionnaire. MF organized the distribution of the questionnaire to the public and wrote the paper, with editing input predominantly from JR and JM. JM statistically analyzed the data. This study was part of MF's student project. All authors contributed to the article and approved the submitted version.

## Funding

Financial support for the project was provided by Norm Mayne, a donor to The University of Queensland, and resource support was provided by the Australian Pet Welfare Foundation.

## Conflict of Interest

JM was employed by Jemora Pty Ltd. The remaining authors declare that the research was conducted in the absence of any commercial or financial relationships that could be construed as a potential conflict of interest.

## Publisher's Note

All claims expressed in this article are solely those of the authors and do not necessarily represent those of their affiliated organizations, or those of the publisher, the editors and the reviewers. Any product that may be evaluated in this article, or claim that may be made by its manufacturer, is not guaranteed or endorsed by the publisher.

## Appendix

**Table A1:** **A–D** Distribution of 1,375 and 3,874 prey animals identified as close to species level as possible reported to have been caught by 261 dogs and 445 cats, respectively, in the 6 months immediately preceding when their owners completed the study questionnaire.

**Mammals**.				
**Mammal category**	**Native to Australia (Yes) or introduced (No)**	**Species**	**Total number caught by the 261 dogs (% of 626 mammals caught by these dogs)**	**Total number caught by the 445 cats (% of 1523 mammals caught by these cats)**
Possum	Yes	Brushtail possum, *Trichosurus vulpecula*	25 (4)	2 (<0.5)
		Possum, *Phalangeriformes*	7 (1)	7 (<0.5)
		Ringtail possum, *Pseudocheirus peregrinus*	10 (2)	8 (1)
Rabbit / hare	No	Hare, *Leupus*	3 (<0.5)	
		Rabbit, Oryctolagus cuniculus	123 (20)	185 (12)
Rodent	No	Black rat, *Rattus rattus*		7 (<0.5)
		Mouse, *Mus musculus*	12 (2)	230 (15)
		Mouse/rat	10 (2)	1 (<0.5)
		Rat, *Rattus*	215 (34)	215 (14)
		Not identifiable		34 (2)
	Unclassified^*^	Mouse	131 (21)	572 (38)
		Mouse/rat		5 (<0.5)
		Rat	60 (10)	209 (14)
		Not identifiable		24 (2)
Bat	Yes	Bat, *Chrioptera*		3 (<0.5)
		Fruit bat, *Pteropodidae*	1 (<0.5)	
		Microbat, Microchiroptera		1 (<0.5)
Domestic	No	Cat, *Felis catus*	6 (1)	
		Dog, *Canis lupus familiaris*	1 (<0.5)	
		Pig, *Sus scrofa domesticus*	4 (1)	
		Not identifiable	1 (<0.5)	
Other	Yes	Agile wallaby, *Macropus agilis*	1 (<0.5)	
		Antechinus, *Antechinus stuartii*		1 (<0.5)
		Bandicoot, *Peramelemorphia*	2 (<0.5)	5 (<0.5)
		Eastern grey kangaroo, *Macropus giganteus*	2 (<0.5)	
		Feathertail glider, *Acrobates pygmaeus*	1 (<0.5)	4 (<0.5)
		Kangaroo, *Macropodidae*	3 (<0.5)	
		Sugar glider, *Petaurus breviceps*		9 (1)
		Swamp wallaby, *Wallabia bicolor*	1 (<0.5)	
		Wallaby, Notamacropus		1 (<0.5)
	No	Fox, Vulpes vulpes	6 (1)	
Mammals total			626 (100)^a^	1,523 (100)^b^

*Percentages are rounded to the nearest whole number and all percentages <0.5%, are presented as <0.5. Species native to Australia are shown as Yes and introduced species are shown as No*.

a *In total, an estimated 1,375 prey animals had been caught by these 261 study dogs in the 6-month period, of which 49% were mammals, 21% birds, 27% reptiles and 7% amphibians*.

b *In total, an estimated 3,874 prey animals had been caught by these 445 study cats in the 6-month period, of which 39% were mammals, 15% birds, 44% reptiles and 2% amphibians*.

* *Unclassified: Unable to be classified as native or introduced*.

**Table A2:** Birds.

**Bird category**	**Native to Australia (Yes) or introduced (No)**	**Species**	**Total number caught by the 261 dogs (% of 284 birds caught by these dogs)**	**Total number caught by the 445 cats (% of 594 birds caught by these cats)**
Columbidae	Yes	Bronzewing pigeon, *Phaphs chalcoptera*		24 (2)
		Crested pigeon, *Ocyphaphs lophotes*	25 (4)	4 (<0.5)
		Dove, *Columbidae*	2 (<0.5)	1 (<0.5)
		Pigeon, *Columbidae*		4 (<0.5)
		Topknot pigeon, *Lopholaimus antarcticus*	2 (<0.5)	
	No	Dove, *Columbidae*		11 (1)
		Pigeon, *Columbidae*	1 (<0.5)	3 (<0.5)
		Rock dove, *Columba livia*	8 (1)	1 (<0.5)
		Spotted dove, *Spilopelia chinensis*	1 (<0.5)	
		Wood pigeon, *Columba palumbus*	2 (<0.5)	
	Unclassifiable	Dove	2 (<0.5)	10 (1)
		Pigeon	15 (2)	24 (2)
		Pigeon/dove	3 (<0.5)	21 (1)
Passerine	Yes	Bee-eater, *Meropidae*		1 (<0.5)
		Bowerbird, *Ptilonorhynchidae*	3 (<0.5)	
		Butcherbird, *Cracticus torquatus*	2 (<0.5)	
		Crow, *Corvus*	1 (<0.5)	
		Currawong, *Strepera*	2 (<0.5)	
		Figbird, *Sphecotheres*		1 (<0.5)
		Finch, *Fringillidae*		1 (<0.5)
		Honeyeater, *Meliphagidae*		10 (1)
		Australian Magpie, *Gymnorhina tibicen*	14 (2)	5 (<0.5)
		Magpie-lark, *Grallina cyanoleuca*	4 (1)	1 (<0.5)
		New Holland honeyeater, *Phylidonyris novaehollandiae*		5 (<0.5)
		Noisy miner, *Manorina melanocephala*	4 (1)	45 (3)
		Pied currawong, *Strepera graculina*	3 (<0.5)	
		Rainbow bee-eater, *Merops ornatus*	2 (<0.5)	
		Red-browed finch, *Neochmia temporalis*		2 (<0.5)
		Silvereye, *Zosterops lateralis*		2 (<0.5)
		Spotted pardalote, *Pardalotus punctatus*		1(<0.5)
		Superb fairywren, *Malurus cyaneus*		16 (1)
		Wattle bird, *Anthochaera*	2 (<0.5)	11 (1)
		Willie wagtail, *Rhipidura leucophrys*		9 (1)
		Yellow rumped thornbill, *Acanthiza chrysorrhoa*		1 (<0.5)
	No	Black starling, *Sturnus vulgaris*		1 (<0.5)
		Blackbird, *Turdus merula*		2 (<0.5)
		Eurasian blackbird, *Turdus merula*		3 (<0.5)
		Indian myna, *Acridotheres tristis*	3 (<0.5)	16 (1)
		Sparrow, *Passeridae*	7 (1)	37 (2)
		Starling, Sturnus vulgaris	15 (2)	16 (1)
	Unclassifiable	Bird	1(<0.5)	2 (<0.5)
		Blackbird	8 (1)	9 (1)
		Finch	11 (2)	9 (1)
		Myna/miner	4 (1)	23 (2)
		Passerine		19 (1)
		Swallow	1 (<0.5)	2 (<0.5)
		Wren		5 (<0.5)
Psittacine	Yes	Australian ringneck, *Barnardius zonarius*	6 (1)	
		Budgerigar, *Melopsittacus undulatus*		1 (<0.5)
		Cockatoo, *Cacatuidae*	1 (<0.5)	
		Eastern rosella, *Platycercus eximius*		8 (1)
		Galah, *Eolophus roseicapilla*		1 (<0.5)
		King parrot, *Alisterus scapularis*	5 (1)	
		Lorikeet, *Loriini*	15 (2)	3 (<0.5)
		Parrot		8 (1)
		Rainbow lorikeet, *Trichoglossus moluccanus*		2 (<0.5)
		Red-rumped parrot, *Psephotus haematonotus*	13 (2)	
		Red-winged parrot, *Aprosmictus erythropterus*		40 (3)
		Rosella, *Platycercus*	13 (2)	51 (3)
		sulfur-crested cockatoo, *Cacatua galerita*	12 (2)	
	Unclassifiable	Parrot	2 (<0.5)	8 (1)
Wild fowl	Yes	Australian wood duck, *Chenonetta jubata*	1 (<0.5)	
		Pacific black duck, *Anas superciliosa*	2 (<0.5)	
		Scrub turkey, *Alectura lathami*	6 (1)	
	No	Peacock, *Pavo cristatus*	1 (<0.5)	
	Unclassifiable	Duck	2 (<0.5)	31 (2)
		Waterhen, Amaurornis phoenicurus		1 (<0.5)
Domestic	No	Chicken, *Gallus domesticus*	13 (2)	2 (<0.5)
		Guinea fowl, *Numididae*	2 (<0.5)	
		Quail, *Coturnix coturnix*	1 (<0.5)	
Other	Yes	Kookaburra, Dacelo	3 (<0.5)	
Unclassifiable	Unclassifiable	Bird	38 (6)	80 (5)
Birds total			284 (100)^a^	594 (100)^b^

a,b *See Table A1 footnotes*.

**Table A3:** Reptiles.

**Reptile category**	**Native to Australia (Yes) or introduced (No)**	**Species**	**Total number caught By the 261 dogs (% of 370 reptiles caught by these dogs)**	**Total number caught by the 445 cats (% of 1691 reptiles caught by these cats)**
Gecko	Yes	Marbled gecko, *Christinus marmoratus*		2 (<0.5)
		Marbled velvet gecko, *Oedura marmorata*,		1 (<0.5)
	No	Asian gecko		3 (<0.5)
		Asian house gecko, *Hemidactylus frenatus*,	7 (1)	143 (9)
		Gecko, *Eublepharis macularius*	20 (3)	
	Unclassifiable	Gecko	1 (<0.5)	38 (2)
Large lizard	Yes	Banded monitor, *Varanus Scalaris*	1 (<0.5)	
		Bearded dragon, *Pogona*	1 (<0.5)	1 (<0.5)
		Blue-tongued skink, *Tiliqua*	63 (10)	3 (<0.5)
		Bobtail skink, *Tiliqua Rugosa*	3 (<0.5)	
		Eastern water dragon, *Intellagama lesueurii*		1 (<0.5)
		Frilled-neck lizard, *Chlamydosaurus kingii*	1 (<0.5)	
		Jacky Dragon, *Amphibolurus muricatus*,		1 (<0.5)
		King's skink, *Egernia kingii*		1 (<0.5)
		Large lizard, *Varanus*	1 (<0.5)	2 (<0.5)
		Lizard, *Lacertilia*	1 (<0.5)	24 (2)
		Skink, *Scincidae*	1 (<0.5)	
		Tata lizard, *Lophognathus gilberti*	2 (<0.5)	
		Water dragon, *Intellagama lesueurii*	17 (3)	31 (2)
		Water monitor, *Varanus salvator*	2 (<0.5)	
		Water skink, *Eulamprys quoyii*		8 (1)
Lizard	Yes	Lizard, *Lacertilia*	35 (6)	14 (1)
Small lizard/skink	Yes	Skink, *Scincidae*		39 (3)
		Lizard, *Lacertilia*		5 (<0.5)
		Small lizard/skink	194 (31)	1,353 (89)
Snake	Yes	Bandy bandy, *Vermicella annulata*		1 (<0.5)
		Blue-bellied black snake, *Pseudechis guttatus*	1 (<0.5)	
		Brown snake, *Pseudonaja*	1(<0.5)	1 (<0.5)
		Brown tree snake, *Boiga irregularis*		1 (<0.5)
		Copperhead snake, *Agkistrodon contortrix*		1 (<0.5)
		Green tree snake, *Dendrelaphis punctualata*		4 (<0.5)
		Red-bellied black snake, *Pseudechis porphyriacus*	2 (<0.5)	1 (<0.5)
		Snake, *Serpentes*	1 (<0.5)	8 (1)
		Tree snake	2 (<0.5)	1 (<0.5)
		Whip snake, *Masticophis*	5 (1)	3 (<0.5)
Other	Yes	Turtle, *Testudines*	3 (<0.5)	
Reptiles total			370 (100)^a^	1,691 (100)^b^

a,b *See Table A1 footnotes*.

**Table A4:** Amphibians.

**Amphibian category**	**Native to Australia (Yes) or introduced (No)**	**Species**	**Total number caught by the 261 dogs (% of 95 amphibians caught by these dogs)**	**Total number caught by the 445 cats (% of 66 amphibians caught by these cats)**
Frog	Yes	Brown tree frog, *Litoria ewingii*	1 (<0.5)	
		Eastern banjo frog, *Limnodynastes dumerilii*	3 (<0.5)	
		Frog, *Anura*	51 (8)	55 (4)
		Great barred frog, *Mixophyes fasiolatus*		2 (<0.5)
		Green tree frog, *Litoria infrafrenata*	27 (4)	2 (<0.5)
		Striped marsh frog, *Limnodynastes peronii*	1 (<0.5)	5 (<0.5)
Toad	No	Toad, *Bufo bufo*	12 (2)	
Unclassifiable	Unclassifiable			2 (<0.5)
Amphibians total			95 (100)^a^	66 (100)^b^

a,b *See Table A1 footnotes*.
