# Supplementary material for: Do Pet Cats Deserve the Disproportionate Blame for Wildlife Predation Compared to Pet Dogs?
Source: Front Vet Sci. 2021 Oct 25;8:731689. doi: 10.3389/fvets.2021.731689 (PMC8572848; doi:10.3389/fvets.2021.731689)
Supplement: Supplementary file 1 [file Table_1.pdf]

Supplementary Material

Table S1: Questionnaire

1. Which age group do you belong to? □ 18 – 24 □ 25 – 34 □ 35 – 44 □ 45 – 54 □ 55+
2. What is your gender? □ Male □ Female
3. In what country do you currently reside?
4. What is your postcode?
5. Which of the following best describes your residence? □ Residence without garden □ Residence with garden □ Farm, acreage or semi-rural □ Other (please describe)

Dogs:

6. How many dogs do you own?
7. To the best of your knowledge, has this dog caught any prey in the last 6 months?
8. In the past month, how many times have you noticed this dog hunt?
9. Please use the following table to provide details about each sort of animals your dog(s) caught in the last 6 months (If your dog(s) has not caught any animals in the last 6 months, please proceed to the next page)

	**Type of animal caught**	**Number caught in the last month**	**Number caught in the last 6 months**	**Please specify which of your dogs caught this/ these animals, if known**	**Is this animal a native species**
Animal 1	Dropdown list:<br>Rodent, for example rat or mouse; Other mammal, for example possum, koala, wallaby; Reptile or amphibian, for example snake, lizard, frog, toad<br>Bird; Other; Unrecognisable	Dropdown list with numbers from 0 to 9+	Dropdown list with numbers from 1 to 9+	Dropdown list:<br>Dog 1<br>Dog 2<br>Don't know<br>More than one of my dogs has caught this animal	Dropdown list:<br>Yes<br>No<br>Don't know
Animal 2					
Animal 3…					

Cats:

10. How many dogs do you own?
11. To the best of your knowledge, has this cat caught any prey in the last 6 months? Yes/ No
12. In the past month, how many times have you noticed this cat hunt?
13. Please complete the following table for each of your cats:
14. Please use the following table to provide details about each sort of animals your cat(s) caught in the last 6 months (If your cat(s) has not caught any animals in the last 6 months, please proceed to the next page)

	**Type of animal caught**	**Number caught in the last month**	**Number caught in the last 6 months**	**Please specify which of your cats caught this/ these animals, if known**	**Is this animal a native species**
Animal 1	Dropdown list:<br>Rodent, for example rat or mouse; Other mammal, for example possum, koala, wallaby; Reptile or amphibian, for example snake, lizard, frog, toad<br>Bird; Other; Unrecognisable	Dropdown list with numbers from 0 to 9+	Dropdown list with numbers from 1 to 9+	Dropdown list:<br>Cat 1<br>Cat 2<br>Don't know<br>More than one of my cats has caught this animal	Dropdown list:<br>Yes<br>No<br>Don't know
Animal 2					
Animal 3..					
